# Comparative Analysis of Treatment Effects of Different Materials on Thin Oil Films

**DOI:** 10.3390/ma18071486

**Published:** 2025-03-26

**Authors:** Xiuli Wu, Bo Zheng, Haiping Dai, Yongwen Ke, Cheng Cai

**Affiliations:** 1 School of Environmental Science and Engineering, Tianjin University, Tianjin 300350, China; 2 College of Urban and Rural Construction, Hebei Agricultural University, Baoding 071001, China; 3 Shandong Jinwei Hairun Special Separation Equipment Co., Ltd., Weifang 261000, China; daihaiping2004@126.com (H.D.); keyongwen@mtshj.com (Y.K.); caicheng@mtshj.com (C.C.)

**Keywords:** thin oil film, super lipophobic and hydrophobic, hollow fiber membrane, separation of oil and water, regeneration

## Abstract

With the continuous and rapid development of global industries, issues such as offshore oil spills, leakage of organic chemicals, and the direct discharge of industrial oily sewage have caused serious damage to the ecological environment and water resources. Efficient oil–water separation is widely recognized as the solution. However, there is an urgent need to address the difficulties in treating thin oil films on the water surface and the low separation efficiency of existing oil–water separation materials. In view of this, this study aims to investigate high-efficiency oil–water separation materials for thin oil films. Four types of oil–water separation materials with different materials are designed to treat thin oil films on the water surface. The effects of factors such as oil film thickness, pressure, and temperature on the oil–water separation performance of these materials are studied. The viscosities of kerosene and diesel oil are tested, and the adsorption and separation effects of the oil–water separation materials on different oil products and oily organic solvents are examined. In addition, the long-term stability of the movable and portable oil–water separation components is verified. The results show that the oil-absorbing sponge-based oil–water separation membrane has an excellent microporous structure and surface roughness, endowing the membrane surface with excellent hydrophobicity and lipophilicity, and exhibiting good oil–water separation performance. The filtration flux of oil increases with the increase in pressure and temperature. It has good adsorption and separation performance for different oil products and oily organic solvents. Moreover, it maintains stable operation performance during the 12-month long-term oil–water separation process for kerosene and diesel oil.

## 1. Introduction

In recent years, with the rapid development of the national industrial manufacturing industry, oil pollution in water bodies has intensified. In addition to oil spills in rivers and the ocean, an increasing amount of oily industrial wastewater generated during the industrial manufacturing process is being discharged into the environment [1]. Polluted oil can form a thin oil film on the water surface that is insoluble in water [2], which not only causes damage to the ecological environment but also has a significant impact on aquatic organisms.

Oily industrial wastewater mainly comes from industrial processes such as petroleum and natural gas, mining, metal processing, chemical engineering, textile printing and dyeing, etc., [3]. Among them, the polluted oil in oily industrial wastewater mainly includes light petroleum hydrocarbons [4] (such as gasoline, kerosene, diesel oil, etc.), heavy petroleum hydrocarbon aromatic hydrocarbons [5], halogenated hydrocarbons [6] (such as benzene, toluene, chloroform, etc.) and other oily organic solvents that are insoluble or hardly soluble in water. The forms of oil in oily wastewater [7] include floating oil (particle size > 100 μm), dispersed oil (particle size 10–100 μm), emulsified oil (particle size < 10 μm), and dissolved oil (particle size < 0.1 μm). Floating oil [8] will form a thin oil film or oil layer on the surface of the water body.

The presence of the thin oil film will seriously affect the gas exchange process of the water body, leading to oxygen deficiency in the water body and causing the death of a large number of aquatic plants and animals. Moreover, the thin oil film will also affect the sunlight transmittance of the water body, weakening the photosynthesis of aquatic plants. In addition, most polluted oils are flammable or toxic substances [9]. They are not only flammable [10] but also can affect human health through biological enrichment [11], causing immeasurable long-term harm. Therefore, the oily wastewater generated in oil spill accidents or industrial processes must be properly treated. Thus, it is very necessary to find a method with high treatment efficiency and remarkable treatment effect.

Traditional treatment methods for thin oil films include biological methods, chemical methods, and physical methods. Biological methods mainly include the microbial metabolism method [11], which uses the metabolic action of oil-degrading microorganisms to convert oil pollution into the organic components of microorganisms or to proliferate into new microorganisms for purification purposes, the activated sludge method [12] that realizes purification by using suspended microbial flocs, and the biofilm treatment method [13] based on aerobic biological treatment. Chemical methods mainly refer to the coagulation/flocculation method, and the added agents as coagulating agents [14], which separates and removes the thin oil film by forming flocs. Compared with biological methods, chemical methods are simple to operate and have low operating costs. However, the oil removal effect is largely restricted by factors such as the type and dosage of the flocculant, the initial concentration of the oil, the temperature of the oily wastewater, and the pH value [15,16,17,18].

Physical methods mainly include the gravity separation method [19] that separates based on the density difference between oil and water, the adsorption method [20] that realizes separation by adsorbing the thin oil film with oil-absorbing materials, and the membrane separation method [21] that selectively separates oily wastewater by virtue of the selective permeability of the membrane. Among them, the gravity separation method and the adsorption method are slow and inefficient [22]. The membrane separation method, with its technical advantages such as energy conservation, no phase change, simple operation, and no secondary pollution, is expected to achieve efficient recovery of thin oil films [23,24,25].

However, membrane materials still face many challenges in practical applications. Polymer membranes have disadvantages such as weak chemical corrosion resistance, poor mechanical properties, and easy contamination [26,27]. While the brittleness, high manufacturing cost, and easy contamination of ceramic membranes are their main challenges [28,29]. In addition, the short membrane life and the trade-off between the permeation flux and the rejection rate are inevitable problems for both polymer membranes and ceramic membranes.

This study aims to develop high-efficiency oil–water separation materials for thin oil films. Four types of materials are designed, and the effects of factors like oil film thickness, pressure, and temperature on their separation performance are investigated. The viscosities of kerosene and diesel, adsorption and separation effects on different oils, and long-term stability of the separation components are also studied.

## 2. Experimental Section

### 2.1. Materials

Pure Kerosene and Pure Diesel: Provided by Sinopec (Beijing, China). Kerosene is a light petroleum hydrocarbon mainly composed of hydrocarbons with carbon numbers typically in the range of C9–C16. It has a relatively low viscosity (1.6 mPa·s at 20 °C), which is beneficial for its flow through separation materials. Diesel, also from Sinopec, is a heavier petroleum hydrocarbon mixture with carbon numbers usually in the range of C10–C22. Its higher viscosity (3.7 mPa·s at 20 °C) compared to kerosene affects its separation performance.

Waste Kerosene and Waste Diesel: Supplied by Tianjin Yahuan Recycling Resources Co., Ltd., (Tianjin, China). Their chemical compositions are similar to pure kerosene and diesel but may contain impurities from industrial use, which could impact the separation process and the performance evaluation of the separation materials.

Red Marker Sudan III: Analytical-grade, provided by Tianjin Comio Chemical Reagent Co., Ltd., (Tianjin, China). It is used to dye kerosene and diesel in the experiments for better visualization of the oil–water separation process. Chemically, Sudan III is a diazo dye.

Distilled Water: Self-made in the laboratory. It serves as the water phase in the oil–water mixture experiments, and its purity is crucial as impurities could interfere with the separation process.

### 2.2. Preparation Process

#### 2.2.1. Preparation of Oil–Water Separation Materials

The experimental materials are four types of oil–water separation materials, which are, respectively: Oil-absorbent cotton (provided by Handan Hengyong Protective and Clean Products Co., Ltd., OAC, Shijiazhuang, China), oil-absorbent cotton-based oil–water separation membrane (prepared in the laboratory, Oil-absorbent cotton impregnated with graphene, OAC-G), PVC-modified oil-absorbent cotton (prepared in the laboratory, Oil absorbent cotton impregnated with PVC and graphene mixture, OAC-P/G), and double-layer oil-absorbent cotton with sponge (provided by Handan Hengyong Protective and Clean Products Co., Ltd., Double oil absorbent cotton with sponge, DOAC-S).

Among them, the oil-absorbent cotton-based oil–water separation membrane is prepared by uniformly coating PVDF nanofibers on the surface of the oil-absorbent cotton using the synchronous electrospinning–electrospraying method. The PVC-modified oil-absorbent cotton is prepared by impregnating the oil-absorbent cotton with a mixture of PVC and graphene using the impregnation method. The specific preparation steps and conditions are described in detail in our previous research [30].

The above four types of oil–water separation materials are, respectively, made into corresponding oil–water separation components, and the specifications of the components are listed in Table 1.

#### 2.2.2. Experimental Facility

**Figure 1:**
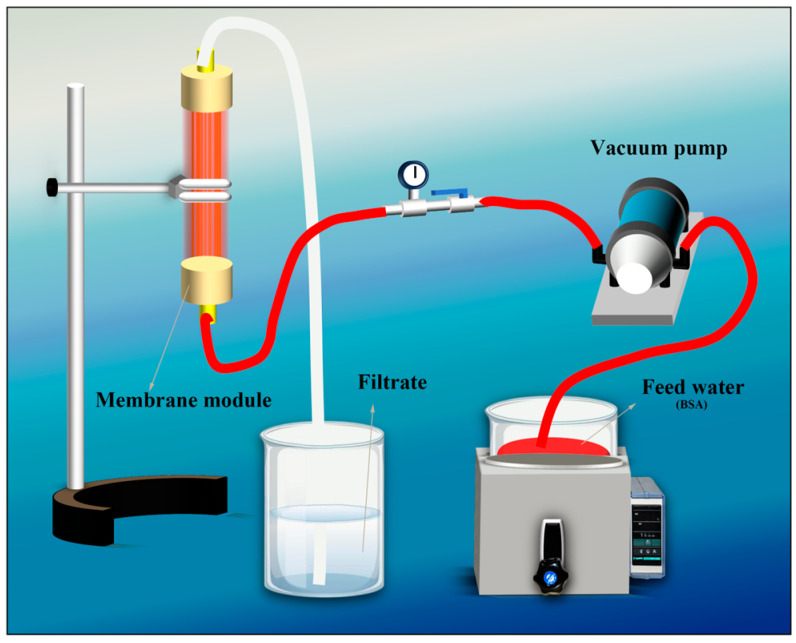
Schematic diagram of oil–water separation test device.

The continuous oil–water separation test device is shown in Figure 1. At room temperature, kerosene and diesel oil dyed with Sudan III are, respectively, mixed with a certain volume of distilled water and placed in a water tank to prepare kerosene (diesel oil)/water mixtures. Different oil-absorbing membrane components are horizontally placed in this water tank so that the components float on the oil–water interface. The interfaces of the components are, respectively, connected to a pressure gauge, a valve, an oil storage tank, and a vacuum pump. The vacuum pump is started, enabling the oil-absorbing membrane components to continuously separate the oil–water mixtures, and the separated oil products are stored in the oil storage tank.

### 2.3. Characterization

#### 2.3.1. Experiment on Flux Test of Oil–Water Separation Materials with Different Materials in Pure Kerosene and Pure Diesel

Under the conditions of a pressure of 0.08 MPa, a temperature of 20 °C, and a filtration time of 2 min, the fluxes of the OAC, OAC-G, OAC-P/G, and DOAC-S oil–water separation materials in pure kerosene and pure diesel oil were tested.

#### 2.3.2. Experiments on the Influence of Thin Oil Film Thickness, Pressure, and Temperature on the Filtration Effects of Oil–Water Separation Materials with Different Materials

Thin Oil Film Thickness: Under the conditions of a pressure of 0.08 MPa and a temperature of 20 °C, the testing device is a water tank with a length × width of 0.29 m × 0.29 m. The OAC, OAC-G, OAC-P/G, and DOAC-S oil–water separation components are, respectively, immersed in the water tank. A certain amount of water is added to the water tank, and then 420.5 mL of kerosene/diesel oil is added. The oil floats on the water to form a thin oil film with a thickness of 5 mm. Every time 84.1 mL of kerosene/diesel oil is filtered out (i.e., the oil amount of a 1 mm thick thin oil film), the time is recorded. Then, the filtration times of the corresponding kerosene/diesel oil when the thicknesses of the thin oil films are 5 mm, 4 mm, 3 mm, 2 mm, and 1 mm are obtained, respectively, and the fluxes at the corresponding thin oil film thicknesses are calculated.

Pressure: An appropriate amount of kerosene (diesel oil)/water mixture is added to the water tank, and the OAC, OAC-G, OAC-P/G, and DOAC-S oil–water separation components are, respectively, immersed in the water tank. At a temperature of 20 °C and a thin oil film thickness of 5 mm, a vacuum pump is used to adjust the filtration pressure. The amounts of oil filtered out within 2 min under the conditions of pressures of 0.05 MPa, 0.06 MPa, 0.07 MPa, 0.08 MPa, and 0.09 MPa are, respectively, recorded, and the membrane filtration fluxes of the four oil–water separation materials for kerosene and diesel oil under different pressures are obtained.

Temperature: An appropriate amount of kerosene (diesel oil)/water mixture is added to the water tank, and the OAC, OAC-G, OAC-P/G, and DOAC-S oil–water separation components are, respectively, immersed in the water tank. Under the conditions of a pressure of 0.08 MPa, an oil film thickness of 5 mm, and a filtration time of 2 min, a water bath pot is used to heat and control the temperature of the oil–water mixture. The volumes of the filtered oil under the conditions of temperatures of 15 °C, 20 °C, 25 °C, 30 °C, 35 °C, and 40 °C are, respectively, measured, and the membrane filtration fluxes of the four oil–water separation materials for kerosene and diesel oil under different temperatures are obtained.

#### 2.3.3. Static Adsorption and Dynamic Separation of Different Oil Products and Oily Organic Solvents

The viscosity of the oil products is tested by a kinematic viscosity tester for petroleum products (Model SYP1003-6B, produced by Shanghai Fuli Instrument Equipment Co., Ltd., Shanghai, China). Select OAC-G as a representative to test the static adsorption and dynamic separation effects of OAC-G on different oil products and oily organic solvents.

The different oil products and oily organic solvents for static adsorption include lubricating oil, hydraulic oil, soybean oil, palm oil, triethyl phosphate, N,N-dimethylacetamide, dimethylformamide, diesel oil, kerosene, and toluene. First, under the conditions of a temperature of 20 °C and an oil film thickness of 1 mm, OAC-G is, respectively, immersed in the above different oil products and oily organic solvents for 120 s. After taking it out, it is placed on a stainless steel filter screen. After 60 s, OAC-G is weighed, and the adsorption capacity is calculated according to the following formula:

$$Q=\frac{W_{b}-W_{a}}{W_{a}}\times 100\%$$

In the formula: *W_a_* and *W_b_*, respectively, represent the mass (g) of OAC-G before and after adsorption, and *Q* is the adsorption capacity (g/g) of OAC-G.

The different oil products and oily organic solvents for dynamic separation include palm oil, xylene, kerosene, diesel oil, butyl acrylate, methyl methacrylate, benzene, toluene, and nonanol. The different oil products and oily organic solvents for dynamic separation testing are all oil–water mixtures. Under the conditions of an oil film thickness of 1 mm, a temperature of 20 °C, a pressure of 0.05 MPa, and a filtration time of 2 min, the fluxes of the OAC-G oil–water separation component for the above different oil products and oily organic solvents are, respectively, tested.

#### 2.3.4. Morphological Characterization

A field emission scanning electron microscope (Model S-4800, Hitachi, Tokyo, Japan) is selected to observe the morphology of the surface and cross-section of the materials. A sample with a length of 6 mm is cut, and the sample is pasted onto the test sample stage with conductive adhesive. After drying and sputtering with gold, the test sample is obtained. At the same time, a sample with a size of 2 mm × 2 mm is pasted onto the sample stage of an atomic force microscope (Model XE-100, Niddatal, Germany), and the test is carried out in the tapping mode. Each sample is measured three times during the test.

#### 2.3.5. XRD Characterization

The surface chemical structure and composition of the materials are characterized by X-ray photoelectron spectroscopy. A uniform sample with a size of 5 mm × 5 mm is cut and fixed on the test sample stage of the energy spectrometer (Thermo Kalpha, Tokyo, Japan). During the test, an Al Kα light source of 1486.6 eV is selected, the binding energy internal standard correction (C1s 284.6 eV) is carried out, and a full spectrum test is performed on the sample. Each type of sample is tested three times.

#### 2.3.6. Specific Surface Area Characterization

The specific surface area is tested by a Brunauer–Emmett–Teller (BET) specific surface area tester (BET-600, Tokyo, Japan). A sample with a size of 1 cm × 1 cm is pasted into the sample introduction unit of the specific surface area tester (SZB-9), and the test is carried out in the automatic mode. Each sample is measured five times during the test.

#### 2.3.7. Static Water Contact Angle Characterization

The surface hydrophilicity of the samples is tested by a semi-automatic contact angle measuring instrument (Model DSA-100, Wiernsheim, Germany). The sample is fixed on a glass slide, and a program is set to drop 1 mL of distilled water onto the surface of the membrane sample. At the same time, the device automatically records the morphological changes in the water droplets and the corresponding contact angle values. Each type of sample is tested five times.

## 3. Results and Discussion

### 3.1. The Viscosities of Kerosene and Diesel Oil

Under the condition of 20 °C, the viscosity test results of kerosene and diesel oil are shown in Table 2. As can be seen from Table 2, the viscosities of kerosene and diesel oil are 1.6 mPa·s and 3.7 mPa·s, respectively. According to relevant reports, the greater the viscosity of the oil product, the greater the transmembrane pressure difference required when it passes through the oil–water separation material. Therefore, under the same pressure, the oil product with a lower viscosity has a higher membrane flux.

### 3.2. Surface Morpography Analysis

**Figure 2:**
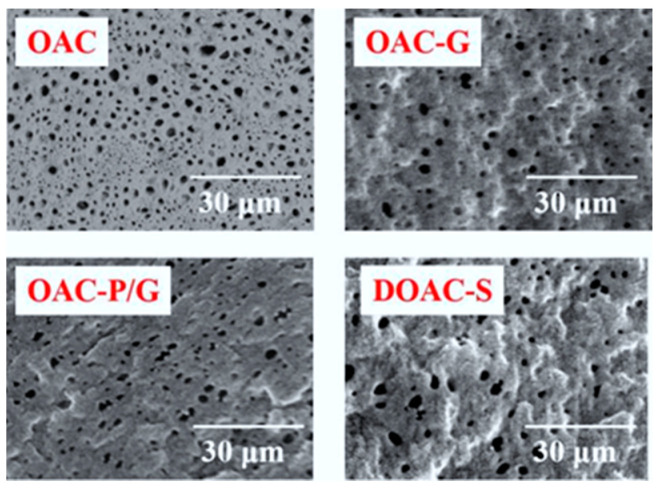
Surface morphologies of oil–water separation materials.

**Figure 3:**
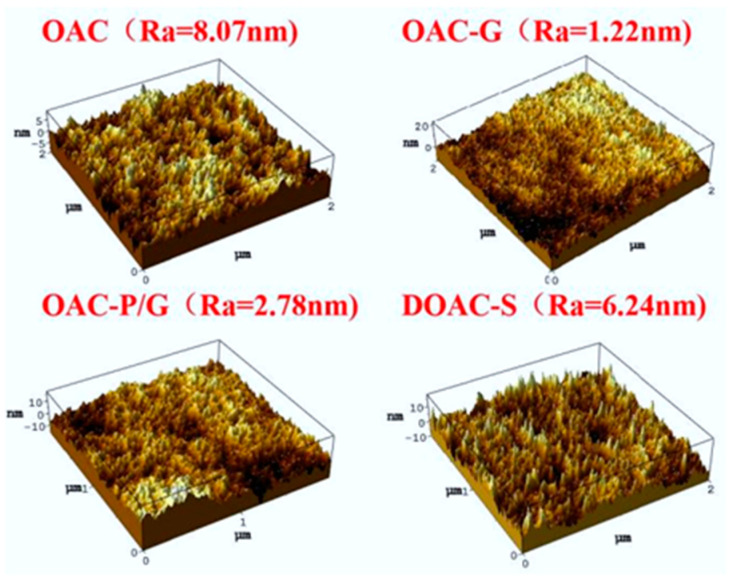
Surface roughness of oil–water separation material.

The surface morphologies and surface roughness of the four oil–water separation materials are shown in Figure 2 and Figure 3, respectively. As can be seen from Figure 2, the surfaces of all four oil–water separation materials exhibit a microporous structure. Among them, the surface microporous structures of the two oil–water separation materials, OAC and DOAC-S, have better penetration and higher porosity. However, after the coating was performed with graphene and PVC/G, the porosity of the surface microporous structures of the oil-absorbent cotton-based oil–water separation material OAC-G and the PVC-modified oil-absorbent cotton oil–water separation material OAC-P/G decreases, and an obvious dense layer appears on the surface, but they still maintain good microporous permeability.

As can be seen from Figure 3, the surface roughness of the two oil–water separation materials, OAC-G and OAC-P/G, is significantly reduced, being 1.22 nm and 2.78 nm, respectively. This is mainly because after the sponge is impregnated with graphene, graphene has good spreadability on the sponge surface, resulting in a better overall flatness of the membrane surface. The surface roughness of the two oil–water separation materials, OAC and DOAC-S, is relatively high, being 8.07 nm and 6.24 nm, respectively.

### 3.3. Surface Structure and Composition Analysis

**Figure 4:**
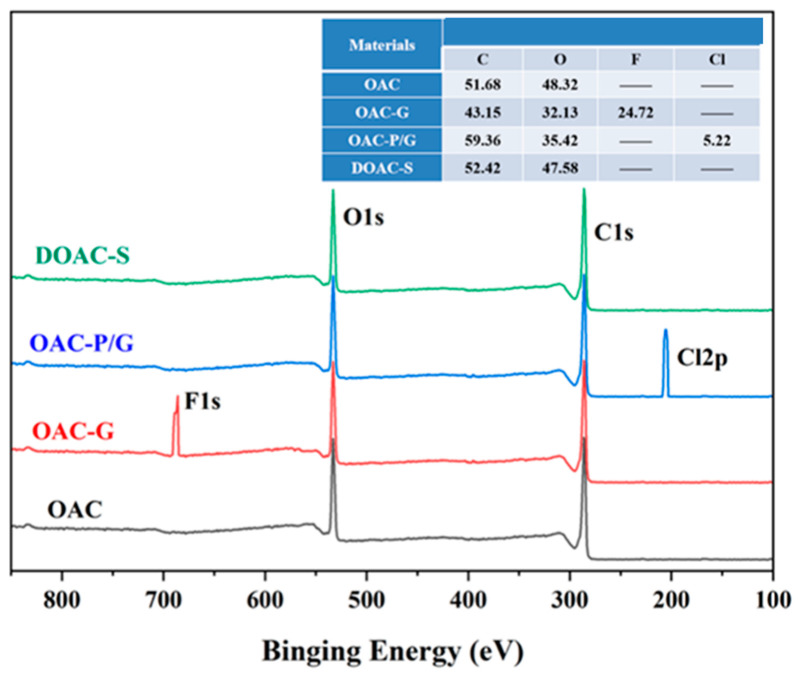
XPS spectrum and element composition of oil–water separation materials.

The XPS analysis results of the surfaces of the four oil–water separation materials are shown in Figure 4. As can be seen from Figure 4, characteristic signal peaks appear at the binding energies of 286 eV and 535 eV on the surfaces of all four oil–water separation materials, corresponding to the characteristic peaks of C 1s and O 1s in the structure of the oil-absorbing cotton OAC, respectively.

Compared with OAC, a characteristic signal peak appears at the binding energy of 687.3 eV for OAC-G, corresponding to the characteristic peak of F 1s in the PVDF structure, and the content of the C element on the surface is significantly reduced. The results confirm that the PVDF nanofibers are successfully coated on the surface of the oil-absorbing cotton by the synchronous electrospinning–electrospraying method.

In addition, a characteristic signal peak appears at the binding energy of 205.9 eV for OAC-P/G, corresponding to the characteristic peak of Cl 2p in the PVC structure. The results confirm that PVC and graphene are successfully coated on the surface of the oil-absorbing cotton by the impregnation method.

### 3.4. Membranes of Kerosene, Diesel Flux

**Figure 5:**
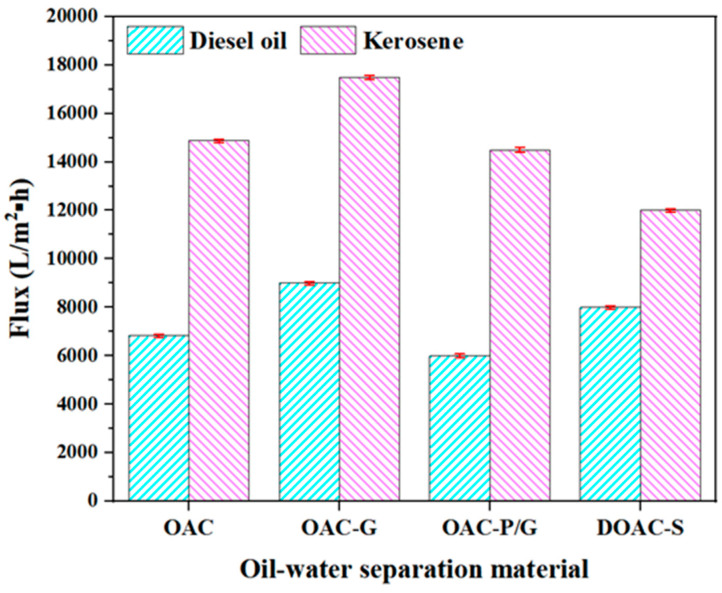
Kerosene, diesel flux of oil–water separation material.

**Figure 6:**
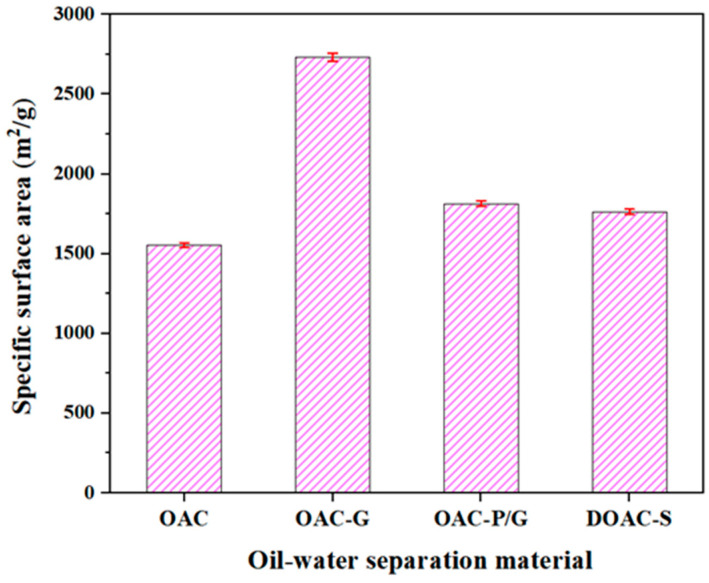
Specific surface area of oil–water separation materials.

The membrane flux of four oil–water separation materials in kerosene and diesel under a vacuum condition of 0.08 MPa is shown in Figure 5. As indicated in Figure 5, during the diesel test, OAC-G demonstrated the highest oil–water separation flux of 9096 L/(m^2^·h), followed by DOAC-S, OAC, and OAC-P/G with fluxes of 8976 L/(m^2^·h), 6827 L/(m^2^·h), and 5989 L/(m^2^·h), respectively. In the kerosene test, OAC-G still showed the highest flux of 17,498.00 L/(m^2^·h), followed by OAC, OAC-P/G, and DOAC-S with fluxes of 14,875 L/(m^2^·h), 14,487 L/(m^2^·h), and 12,010 L/(m^2^·h), respectively. It is evident that the highest oil–water separation flux for diesel is significantly lower than that for kerosene when using the same oil–water separation material, OAC-G. For OAC-G, the flux in the diesel test is 9096 L/(m^2^·h), while in the kerosene test, it reaches 17,498.00 L/(m^2^·h). This large disparity can likely be attributed to the differences in the physical properties of diesel and kerosene. Kerosene generally has a lower viscosity and surface tension compared to diesel. These properties affect the flowability of the fluids through the oil–water separation materials. With a lower viscosity and lower surface tension, kerosene can more easily pass through the pores of the material, resulting in a higher flux. In contrast, the relatively higher viscosity and surface tension of diesel may impede its flow through the material, leading to a lower separation flux. Additionally, the chemical composition of diesel and kerosene might also have an impact on their interaction with the oil–water separation materials, potentially influencing the separation efficiency and flux. This indicates that when selecting oil–water separation materials for different oil types, the physical and chemical properties of the oils need to be carefully considered to optimize the separation performance. Comparison shows that OAC-G exhibited superior separation performance for both kerosene and diesel compared to other materials. This may be attributed to the modified oil-absorbing cotton-based material having a smoother surface and larger specific surface area (Figure 6, OAC-G specific surface area is 2733 m^2^/g, while OAC-P/G, DOAC-S, and OAC show values of 1815 m^2^/g, 1763 m^2^/g, and 1554 m^2^/g, respectively), leading to better wettability towards kerosene and diesel and thus faster filtration rates.

### 3.5. Influence of Different Thin Oil Film Thicknesses on the Separation Performance of Four Oil–Water Separation Materials

**Figure 7:**
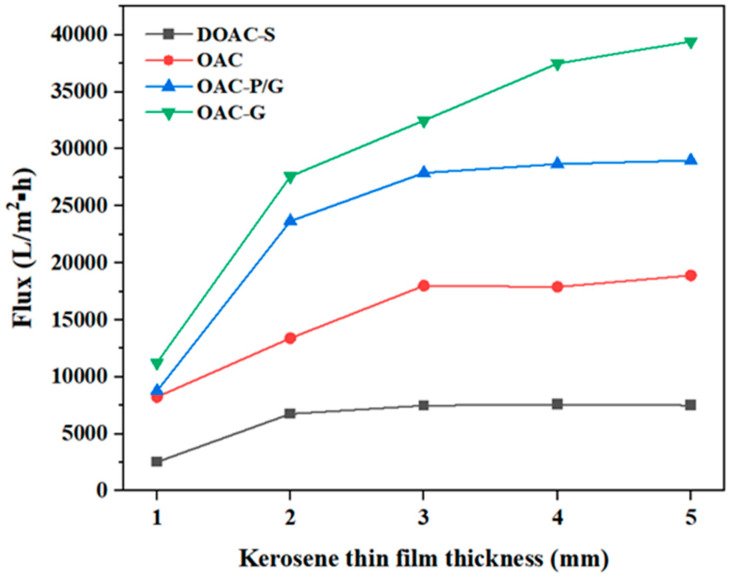
Flux of oil–water separation materials under different kerosene thin film thicknesses.

**Figure 8:**
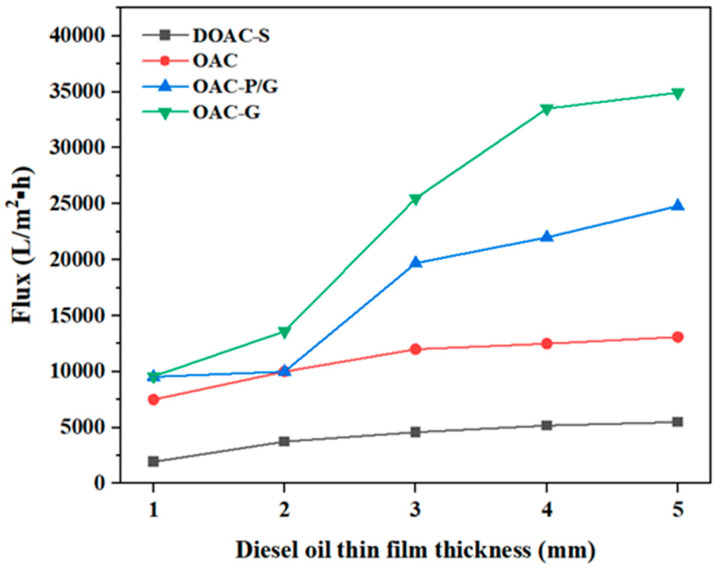
Flux of oil–water separation materials under different thin film thicknesses of diesel oil.

The influence of the thin oil film thicknesses of kerosene and diesel on the separation performance of the four oil–water separation materials is shown in Figure 7 and Figure 8, respectively. As can be seen from the figures, the separation ability of the four oil–water separation materials for kerosene is higher than that for diesel. When the thin oil film thickness is less than 2 mm, the fluxes of both kerosene and diesel are relatively small. Especially when the thin oil film thickness is less than or equal to 1 mm and during the final process of recovering the oil slick, the fluxes decrease significantly. This may be because when the thin oil film is too thin, the effective area of the oil–water separation material immersed in the thin oil film becomes smaller, resulting in a decrease in the flux.

**Figure 9:**
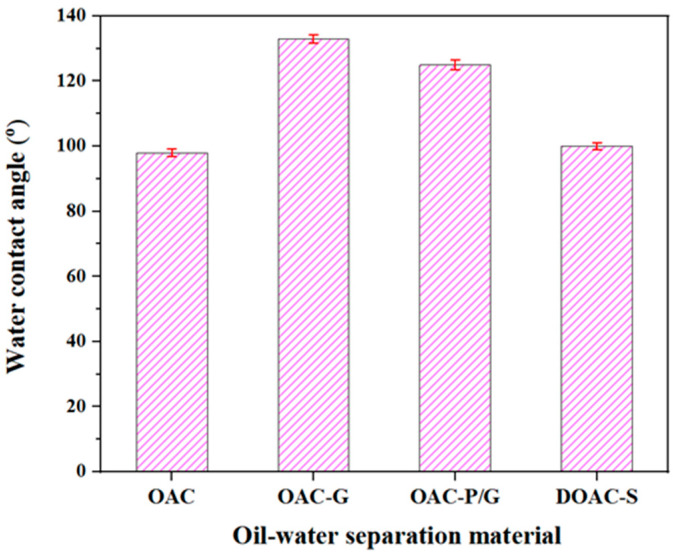
Water contact angle of different materials.

When the thin oil film thickness is between 3 and 5 mm, the membrane fluxes of the four oil–water separation materials gradually increase. OAC-G has the highest flux, with membrane fluxes for kerosene and diesel of 39,431 L/(m^2^·h) and 3375 L/(m^2^·h), respectively. This may be because after the oil-absorbing cotton is impregnated with graphene, its hydrophobicity is improved (Figure 9 shows that the water contact angle of OAC-G is 133°, while the water contact angles of the other three materials, OAC-P/G, DOAC-S, and OAC, are 125°, 100°, and 98°, respectively), and its lipophilicity is enhanced. Therefore, its flux is higher than that of the oil–water separation materials made of other materials.

When comparing the oil–water separation fluxes of diesel and kerosene for DOAC-S and OAC, despite the fact that their fluxes are relatively small overall, there are still notable differences between the two oils. For these two materials, the flux for kerosene is higher than that for diesel. This can be related to the inherent properties of diesel and kerosene. As mentioned earlier, kerosene typically has lower viscosity and surface tension compared to diesel. Even for materials like DOAC-S and OAC which are described as thicker and more compact, the more fluid nature of kerosene enables it to pass through the membranes more easily, thus resulting in a higher flux. On the other hand, the higher viscosity of diesel makes it more resistant to flow through the relatively restricted pores of these thicker and more compact materials, leading to a lower separation flux. This further emphasizes that the physical characteristics of the oils play a crucial role in determining the oil–water separation flux, and for materials with certain structural properties (such as being thick and compact), the differences in oil properties have a more pronounced impact on the separation performance.

In the case of OAC-G, although it has relatively high fluxes for both oils, the difference in flux between kerosene and diesel still exists, following the same trend of kerosene having a higher flux. This suggests that regardless of the overall performance level of the material, the nature of the oil being separated significantly influences the efficiency of the oil–water separation process. It also implies that when applying these materials in practical scenarios, choosing the appropriate material based on the specific oil type is essential for achieving optimal separation results.

### 3.6. Influence of Different Pressures on the Filtration Performance of the Oil-Absorbent Cotton-Based Oil–Water Separation Material

**Figure 10:**
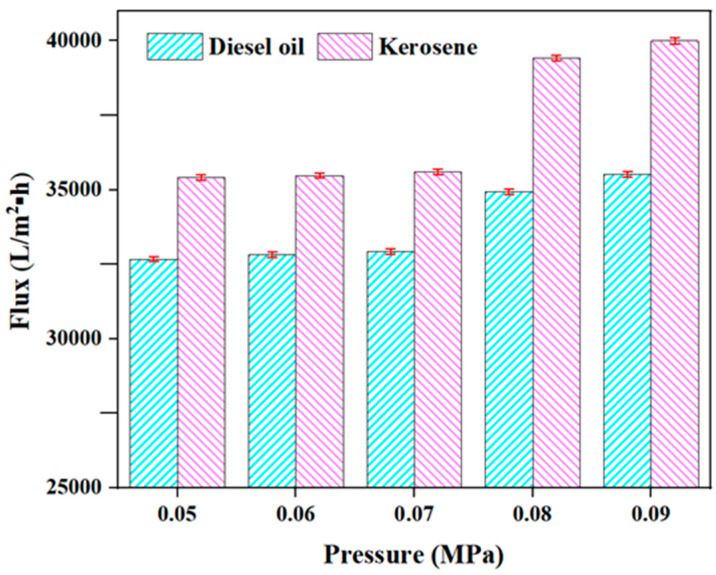
OAC-G fluxes of kerosene and diesel at different pressures.

In Figure 10, it could be seen that “The membrane flux of OAC-G for kerosene increases from 32,695 L/(m^2^·h) to 35,520 L/(m^2^·h), and the membrane flux for diesel increases from 35,415 L/(m^2^·h) to 39,988 L/(m^2^·h)”. However, according to the bar chart (Figure 10), the initial flux (at 0.05 MPa) for kerosene is approximately 35,500 L/(m^2^·h), and for diesel it is approximately 32,500 L/(m^2^·h). So, the initial flux values in the text are reversed for kerosene and diesel compared to the figure. The text correctly states that “as the pressure increases, the membrane fluxes of OAC-G for both kerosene and diesel gradually increase”. This is consistent with the bar chart in Figure 10, where we can see that as the pressure increases from 0.05 MPa to 0.09 MPa, the height of both the pink (kerosene) and blue (diesel) bars increases, indicating an increase in flux. In summary, the text has incorrect initial flux values for kerosene and diesel when compared to Figure 10, but the description of the flux-increasing trend with increasing pressure is consistent with the figure.

### 3.7. Influence of Different Temperatures on the Filtration Performance of the Oil-Absorbent Cotton-Based Oil–Water Separation Material

**Figure 11:**
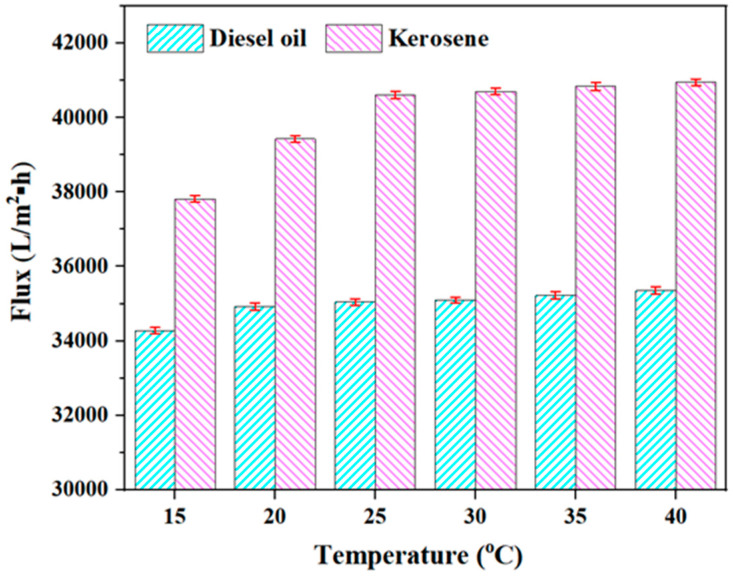
OAC-G fluxes of kerosene and diesel at different temperatures.

Figure 11 shows the filtration flux results of OAC-G for filtering kerosene and diesel under the conditions of 15 °C, 20 °C, 25 °C, 30 °C, 35 °C, and 40 °C. As can be seen from Figure 11, with the gradual increase in temperature, the membrane fluxes of OAC-G for both kerosene and diesel gradually increase. The membrane flux of OAC-G for kerosene increases from 37,815 L/(m^2^·h) to 40,950 L/(m^2^·h), and the membrane flux for diesel increases from 34,275 L/(m^2^·h) to 35,350 L/(m^2^·h). The reason for this phenomenon is that as the temperature rises, the viscosity of the oil products decreases, the membrane mass transfer resistance decreases, and it is easier for the oil products to pass through the pores of OAC-G.

### 3.8. Analysis of the Recovery Tests of Different Oil Products and Oily Organic Solvents

**Figure 12:**
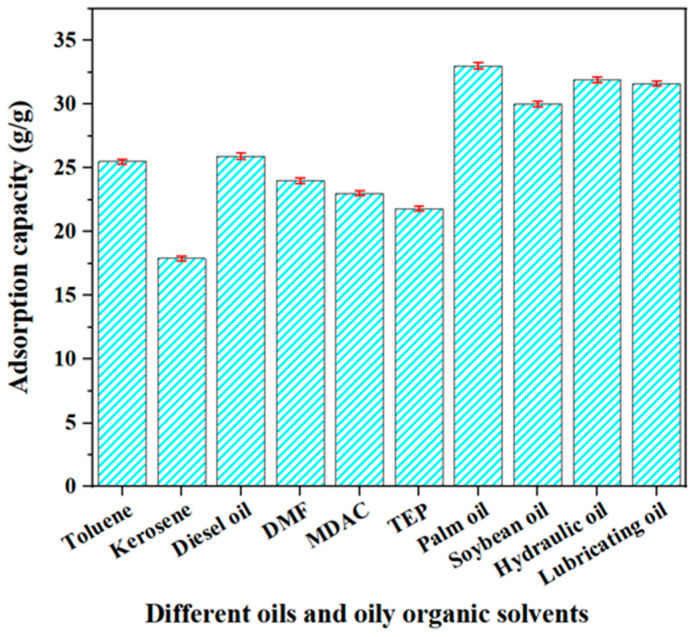
Static adsorption capacity of OAC-G for different oils and organic solvents.

The leakage of different oil products and oily organic solvents can have a significant impact on both the ecological environment and the human environment. Considering that the oil–water separation membrane has an excellent recovery effect on kerosene and diesel, the performance of the OAC-G material in adsorbing and separating different oil products and oily organic solvents on the water surface is verified and evaluated. All the different oil products and oily organic solvents used in the experiment are lightweight and can float on the water surface. The results of the static adsorption experiment are shown in Figure 12.

As can be seen from Figure 12, OAC-G has a very good adsorption effect on diesel, kerosene, and other different oil products and oily organic solvents. Among them, the adsorption capacity for palm oil is the highest, which is 33 g/g. It also has a good adsorption effect on diesel and kerosene, which are 25.9 g/g and 17.9 g/g, respectively. This may be because OAC-G has a large number of microporous structures. After being modified by graphene, its hydrophobicity is enhanced and its lipophilicity is greatly improved, which is consistent with the previous analysis results of the membrane surface morphology and hydrophilic performance.

**Figure 13:**
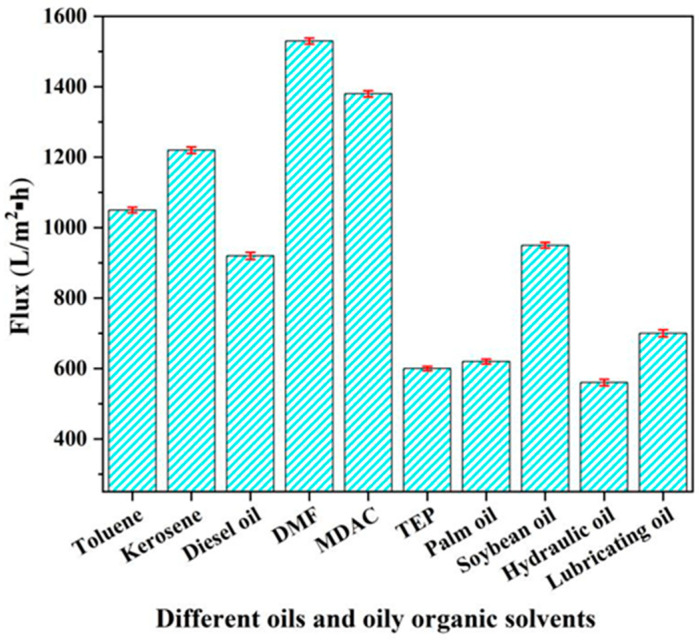
Dynamic membrane flux of OAC-G for different oils and oily organic solvents.

After the static adsorption test is completed, the OAC-G component is connected to the power system for the dynamic separation experiment, and the results are shown in Figure 13. As can be seen from Figure 13, the OAC-G component also has a very good dynamic separation effect on different oil products and oily organic solvents. The one with the highest membrane flux is dimethylformamide, and its membrane flux is 1530 L/(m^2^·h). Followed by dimethylacetamide, kerosene, toluene, soybean oil, diesel, lubricating oil, palm oil, triethyl phosphate, and hydraulic oil. The one with the lowest flux is palm oil. In addition to the structure of the component itself, the main factors affecting the filtration flux may also include the molecular structure, polarity, and viscosity of the oil products and oily organic solvents.

### 3.9. Analysis of the Long-Term Operation Effect of OAC-G Material

**Figure 14:**
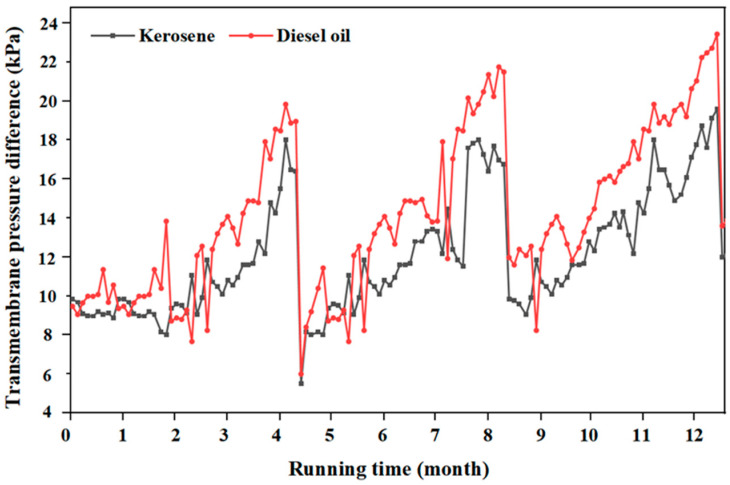
The OAC-G material changes the transmembrane pressure difference in kerosene and diesel oil for 12 consecutive months.

Based on the above analysis, considering the long-term performance of the membrane, under the condition of setting a constant flux of 35,000 L/(m^2^·h) for the membrane unit, continuous and stable operation of separating kerosene and diesel using OAC-G was carried out for 12 months, respectively. The cleaning cycle was 4 months, and the cleaning process was a combined cleaning scheme of first soaking and cleaning with 1000 mg/L NaOH and then soaking and cleaning with 1000 mg/L HCl. The transmembrane pressure difference data of the two membrane units for separating kerosene and diesel were monitored and recorded. The results are shown in Figure 14. The peaks in the curve represent the pressure difference state of the membrane after being contaminated, and the troughs represent the state of the membrane pollution recovery and the decrease in the pressure difference after cleaning.

As can be seen from Figure 14, when the flux remains unchanged, the membrane oil filtration resistance continuously increases, and the transmembrane pressure difference also continuously increases. When it increases to 18–22 kPa at around the 4th month, it indicates that the membrane has been contaminated. Then, the first off-line chemical cleaning was carried out on the two groups of membranes, respectively. After cleaning, the transmembrane pressure difference in the kerosene oil–water separation system decreased from 17.9 kPa to 5.2 kPa, and the transmembrane pressure difference in the diesel oil–water separation system decreased from 19.6 kPa to 5.8 kPa. Subsequently, two off-line chemical cleanings were carried out, respectively, after the 8th month and the 12th month. From the changes in the transmembrane pressure difference curve data before and after cleaning, it can be seen that the SC membrane achieved a good flux recovery effect after cleaning. At the same time, it can also be seen from the figure that the overall transmembrane pressure difference before and after cleaning shows a slightly increasing trend.

## 4. Conclusions

In this study, four types of oil–water separation materials were used to treat thin oil films on the water surface. The effects of factors such as oil film thickness, pressure, and temperature on the filtration performance of the oil–water separation materials were investigated. By testing the viscosities of kerosene and diesel, as well as the adsorption and separation effects of the oil–water separation materials on different oil products and oily organic solvents, the long-term stability of the movable and portable components was verified.

The results show that the oil-absorbent cotton-based oil–water separation material has an excellent microporous structure and membrane surface roughness, endowing the membrane surface with excellent hydrophobicity and lipophilicity. It has good oil–water separation performance. The filtration flux of oil increases with the increase in pressure and temperature, and it has good adsorption and separation performance for different oil products and oily organic solvents. At the same time, it maintains stable operation performance during the 12-month long-term oil–water separation process for kerosene and diesel.

The research results can effectively address the existing problems of the difficulty in treating thin oil films on the water surface and the low separation efficiency of current oil–water separation materials, and achieve efficient oil–water separation of thin oil films.

## Figures and Tables

**Table 1:** Specifications of four oil–water separation components.

Code	Specification
Component length (m)	0.08
Component diameter (m)	0.014
Component area (m^2^)	0.0035

**Table 2:** The viscosity of different oils.

Oil Type	Viscosity (mPa·s)
Kerosene	1.6
Diesel	3.7

## Data Availability

The original contributions presented in this study are included in the article. Further inquiries can be directed to the corresponding authors.
